# A microRNA Transcriptome-wide Association Study of Prostate Cancer Risk

**DOI:** 10.3389/fgene.2022.836841

**Published:** 2022-03-30

**Authors:** Nicholas B. Larson, Shannon K. McDonnell, Zachary Fogarty, Yuanhang Liu, Amy J. French, Lori S. Tillmans, John C. Cheville, Liang Wang, Daniel J. Schaid, Stephen N. Thibodeau

**Affiliations:** ^1^ Department of Quantitative Health Sciences, Mayo Clinic, Rochester, MN, United States; ^2^ Department of Laboratory Medicine and Pathology, Mayo Clinic, Rochester, MN, United States; ^3^ Department of Tumor Biology, H. Lee Moffitt Cancer Center, Tampa, FL, United States

**Keywords:** prostate cancer, eQTL, microRNA, expression, TWAS

## Abstract

Large genome-wide association studies have identified hundreds of single-nucleotide polymorphisms associated with increased risk of prostate cancer (PrCa), and many of these risk loci is presumed to confer regulatory effects on gene expression. While eQTL studies of long RNAs has yielded many potential risk genes, the relationship between PrCa risk genetics and microRNA expression dysregulation is understudied. We performed an microRNA transcriptome-wide association study of PrCa risk using small RNA sequencing and genome-wide genotyping data from N = 441 normal prostate epithelium tissue samples along with N = 411 prostate adenocarcinoma tumor samples from the Cancer Genome Atlas (TCGA). Genetically regulated expression prediction models were trained for all expressed microRNAs using the FUSION TWAS software. TWAS for PrCa risk was performed with both sets of models using single-SNP summary statistics from the recent PRACTICAL consortium PrCa case-control OncoArray GWAS meta-analysis. A total of 613 and 571 distinct expressed microRNAs were identified in the normal and tumor tissue datasets, respectively (overlap: 480). Among these, 79 (13%) normal tissue microRNAs demonstrated significant cis-heritability (median cis-h2 = 0.15, range: 0.03–0.79) for model training. Similar results were obtained from TCGA tumor samples, with 48 (9%) microRNA expression models successfully trained (median cis-h2 = 0.14, range: 0.06–0.60). Using normal tissue models, we identified two significant TWAS microRNA associations with PrCa risk: over-expression of mir-941 family microRNAs (P_TWAS_ = 2.9E-04) and reduced expression of miR-3617-5p (P_TWAS_ = 1.0E-03). The TCGA tumor TWAS also identified a significant association with miR-941 overexpression (P_TWAS_ = 9.7E-04). Subsequent finemapping of the TWAS results using a multi-tissue database indicated limited evidence of causal status for each microRNA with PrCa risk (posterior inclusion probabilities <0.05). Future work will examine downstream regulatory effects of microRNA dysregulation as well as microRNA-mediated risk mechanisms via competing endogenous RNA relationships.

## Introduction

Prostate cancer (PrCa) is a highly heritable complex disease, and large genome-wide association studies (GWAS) and meta-analyses have identified hundreds of single-nucleotide polymorphisms (SNPs) associated with increased risk of PrCa (Gudmundsson et al., 2007; Yeager et al., 2007; Salinas et al., 2008; Schaid et al., 2010; Kote-Jarai et al., 2011; Akamatsu et al., 2012; Cheng et al., 2012; Al Olama et al., 2013; Kote-Jarai et al., 2013; Al Olama et al., 2014; Hazelett et al., 2014; Yang et al., 2014; Amin Al Olama et al., 2015; Hoffmann et al., 2015; Teerlink et al., 2016). However, the majority of these risk loci are intergenic and few causal genes for PrCa have been explicitly identified (Eeles et al., 2014). For many identified loci, the underlying causal variants are presumed to confer regulatory effects on expression of nearby genes. Follow-up expression quantitative trait locus (eQTL) studies have begun to explore putative regulatory targets of PrCa risk SNPs (Thibodeau et al., 2015; DeRycke et al., 2019). Recent methodological advancements in colocalization of disease-associated SNPs and eQTLs have yielded powerful transcriptome-wide association study (TWAS) approaches to disease-gene discovery, which have been highly successfully in translating single-SNP GWAS hits into gene-level associations with PrCa risk (Mancuso et al., 2018; Liu et al., 2022).

Most eQTL-based functional studies of PrCa risk loci have focused primarily on protein-coding genes, and classes of non-coding RNA transcripts remain understudied. An important class of small non-coding RNAs is microRNAs (miRNAs), which contribute to transcriptome regulation via post-transcriptional silencing mechanisms of translational repression, and RNA degradation. MiRNAs are initially expressed as long precursor primary miRNA (pri-miRNA) transcripts before multiple post-processing steps yield short mature miRNAs typically 20–22 nucleotides in length. These mature miRNAs in turn bind to miRNA response elements (MREs) located primarily in the 3-prime untranslated regions (3′UTRs) of target RNA transcripts. There is a large body of evidence demonstrating the roles of miRNAs in cancer (Peng and Croce, 2016). Specific to PrCa, upregulation of miR-124 has been shown to repress PrCa growth and inhibit invasion of PrCa tumor cells (Kang et al., 2014), and circulating miRNA expression profiles demonstrate diagnostic value for aggressive PrCa (Alhasan et al., 2016). Previous multi-omic analyses in the Cancer Genome Atlas (TCGA) prostate adenocarcinoma (PRAD) samples also identified associations of unsupervised miRNA expression clusters and tumor Gleason score (Cancer Genome Atlas Research, 2015).

Recent eQTL studies have also shown pri-miRNA transcripts are subject to the same *cis*-acting SNP regulatory effects as other long RNAs (Huan et al., 2015), and PrCa risk SNPs may confer systemic impacts on the prostate transcriptome through miRNA expression dysregulation. However, large miRNA expression datasets in relevant tissue types are necessary for eQTL-based disease gene discovery, and such data for normal prostate tissue are lacking. In this study, we present data from a large miRNA sequencing dataset in normal prostate tissue, explore *cis*-based predictive modeling of miRNA expression, and evaluate potential associations between PrCa risk genetics and expression dysregulation of miRNAs by performing a miRNA-based TWAS (miTWAS) of expressed miRNAs in normal and tumor prostate tissue, which may respectively reveal key risk gene associations relevant to various stages of tumorigenesis.

## Methods

### Normal Prostate Tissue Samples

Information on study tissue sample selection has been described in greater detail elsewhere (Larson et al., 2015; Thibodeau et al., 2015). Briefly, normal prostate tissue was acquired from an archive collection of fresh frozen material obtained from patients with either radical prostatectomy or cystoprostatectomy. After rigorous evaluation of sample tissue quality, DNA was extracted using the Puregene tissue extraction while RNA was extracted using the QIAGEN miRNeasy Mini Kit and the QIAcube instrument. Informed consent was obtained from all subjects, and the study was approved by the Mayo Clinic Institutional Review Board.

### Normal Prostate eQTL Dataset

Descriptions on genome-wide genotyping and RNA sequencing (RNA-Seq) have been provided elsewhere (Larson et al., 2015; Thibodeau et al., 2015; DeRycke et al., 2019), and these data are publicly available on dbGaP (accession: phs000985. v1. p1). Briefly, genome-wide genotyping was performed using the Illumina Infinium 2.5 M bead arrays (Illumina, San Diego, CA). Quality control excluded variants with low call rate (<95%) and Hardy–Weinberg equilibrium testing (P < 1E-05). Untyped and missing autosomal and chrX SNP genotypes were imputed via SHAPEIT (Delaneau et al., 2013) and IMPUTE2(Howie et al., 2012) using the hg19/GRCh37 1,000 Genomes Phase I reference panel. Imputation quality was assessed using the dosage r^2^ metric calculated by BEAGLE v3 utilities (Browning and Browning, 2009), and SNPs with an r2 ≤0.3 were excluded from further analysis. After imputation and quality filtering, a total of 9,915,470 variants were available for analysis.

RNA libraries for sequencing were prepared using the TruSeq RNA Sample Prep Kit v2. Paired-end sequencing was performed on an Illumina HiSeq 2000 using TruSeq SBS sequencing kit version 3 and HCS v2.0.12 data collection software. A minimum of 50 million total reads per sample was required for analysis; 234 samples with <50 million total reads were re-sequenced and BAM files were merged if no quality issues were identified. RNA-seq data were processed using the MAP-R-Seq pipeline (Kalari et al., 2014) and gene counts were generated based on ENSEMBL release 78 gene annotation. Conditional quantile normalization (Hansen et al., 2012) was applied to account for GC-bias and sequencing depth. Normalized expression values were then processed using probabilistic estimation of expression residuals (PEER) (Stegle et al., 2012), adjusting for known sample histologic characteristics of percent lymphocytic population and percent epithelium present. PEER residuals were used as the final expression values for all subsequent analyses.

### Normal Prostate Small RNA Sequencing

Among the N = 471 Mayo normal tissue samples with available genome-wide genotyping and RNA-Seq data, we identified N = 444 total samples with sufficient residual material for small RNA extraction. RNA was extracted using the Qiagen miRNAeasy Mini Kit and the QIAcube instrument. Small RNA sequencing was performed on an Illumina HiSeq 4,000 instrument using NEBNext® Multiplex Small RNA Library Prep Kit, multiplexing up to 48 samples per lane. Sequencing output was processed using the CAP-Mirseq (Sun et al., 2014) bioinformatics pipeline using miRBase v21 (Griffiths-Jones et al., 2006) miRNA reference. An in-house developed RNA-Seq QC (RNASEQQC) pipeline was then applied to identify potential issues based on measures of post-normalization quality (Mahoney et al., 2013).

Mature miRNAs were identified as expressed if ≥ 5 aligned reads were identified in >25% of samples, and raw expression counts for expressed miRNAs were normalized using trimmed mean of M (TMM) normalization (Robinson and Oshlack, 2010). Leading principal components from the genotyping data did not indicate associations with miRNA expression levels (min P > 1e-05). Normalized expression values were then processed using probabilistic estimation of expression residuals (PEER) (Stegle et al., 2012), adjusting for known sample histologic characteristics of percent lymphocytic population, and percent epithelium present. PEER residuals were used as the final expression values for all subsequent analyses.

### TCGA Tumor Data

To additionally explore associations in cancer tissue and provide potential replication of normal tissue discoveries, we also considered miTWAS analyses using miRNA expression data from TCGA prostate adenocarcinoma (PRAD) tumor tissue. Information on the sample preparation and small RNA sequencing are presented in greater detail elsewhere (Chu et al., 2016). To reduce underlying technical differences between our normal tissue miRNA expression dataset and the TCGA small RNA sequencing results, TCGA PRAD small RNA-Seq BAM files were downloaded from the Genomic Data Commons (GDC), of which N = 411 were identified to correspond to tumor tissue. BAM files were reverted to unaligned FASTQ files and reprocessed using the identical miRNA bioinformatics workflow described above. With respect to PEER analyses, sample estimated tumor percentage was used as an adjusting covariate.

Raw genome-wide genotyping Birdseed files from the Affymetrix 6.0 genotyping panel were similarly retrieved from the GDC online portal for all TCGA PRAD samples. We identified one unique non-tumor file per subject (either whole blood or normal tissue), with preferential selection of whole blood data where available. This yielded N = 496 samples (439 blood, 57 normal tissue), which were processed for genotype calling using a genotype confidence threshold of 0.5. Genotype data were then further processed for SNP- and sample-level QC as similarly described for the normal tissue genotyping data. Based on output from STRUCTURE, N = 415 samples were identified that corresponded to subjects of European descent (Caucasian probability>0.8). The resulting genome-wide genotyping dataset was then imputed using the Michigan Imputation Server (Das et al., 2016) based on the hg19/GRCh37 Haplotype Reference Consortium (McCarthy et al., 2016) hrc. r1.1.2016 reference panel, with phasing performed using Eagle v2.4.

### miRNA Transcriptome-wide Association Study

Our miTWAS was based on PrCa case-control GWAS meta-analysis summary statistics from the PRACTICAL Consortium (Schumacher et al., 2018), downloaded from the PRACTICAL website (http://practical.icr.ac.uk). For the respective Mayo and TCGA miRNA eQTL datasets, genetically-regulated expression (GReX) prediction models were trained using the TWAS software FUSION (Gusev et al., 2016). Eligibility for GReX model training was determined based on a cis-heritability (cis-h^2^) test *p*-value <0.01 and corresponding cis-h^2^ estimate >0, and training was performed using elastic-net with a 1 Mb buffer for cis-variant inclusion, with all other settings left at default values. Similar methods were used to train GReX models for all expressed long RNAs (coding and non-coding) from the previously mentioned Mayo normal tissue RNA-Seq data for purposes of comparison and downstream TWAS finemapping at significant miTWAS loci. Expressed miRNAs overlapping the major histocompatibility complex (MHC) region, defined as hg19/GRCh37 positions chr6:28,477,797–33,448,354, were excluded from analysis due to the complex LD structure in this region.

FUSION was then used in conjunction with the PRACTICAL PrCa risk GWAS meta-analysis summary statistics to generate miTWAS results for all expressed miRNAs that had successfully trained GReX models. The corresponding LD reference was derived from the normal tissue genotyping dataset previously described. Results were declared statistically significant separately for normal and tumor tissue analyses based on a Benjamini–Hochberg false discovery rate (FDR) criterion of FDR<0.05.

### TWAS Finemapping

Significant non-causal TWAS results may cluster at a given risk region due to LD and/or shared regulatory regions. TWAS finemapping methods can help resolve which gene(s) may be causal by simultaneously evaluating multiple genes in a given region. For significant normal tissue miTWAS associations, we applied the TWAS finemapping software FOCUS(Mancuso et al., 2019) using a customized version of the FOCUS multi-tissue GReX database (focus.db, downloaded November 2020) that was augmented by additionally including all miRNA and mRNA GReX models derived from the Mayo normal tissue dataset. FOCUS was otherwise run under default settings per chromosome, which included output for marginal posterior inclusion probabilities and inclusion in the 90% credible set.

## Results

Small RNA sequencing yielded a median total throughput of 8.0 million reads per sample, with a range of 1.2–22.8 million reads. Of the N = 444 normal tissue samples processed for small RNA sequencing, 441 (99.3%) passed sample-level quality control thresholds for analysis. There was a median of 4.1 million mature miRNA-aligned reads per sample (range: 0.3–15.5 million; Supplementary Figure S1). A total of 613 distinct mature miRNAs were identified as expressed per our criterion of ≥5 aligned reads in >25% of samples. Among the 411 TCGA PRAD tumor samples with reprocessed miRNA expression data, no QC sample exclusions were made, and there was a median 7.5 (range 1.9–34.6 million; Supplementary Figure S2) million miRNA-aligned reads. A total of 571 distinct expressed miRNAs were identified (overlap with normal prostate expression data: 480). A total of 3 and 2 expressed miRNAs overlapped the MHC region in the normal and tumor tissue datasets, respectively, and were excluded from further analysis.

### MiRNA GReX Models

Among the remaining 610 non-MHC miRNAs expressed in normal prostate tissue, cis-h^2^ testing via FUSION was successfully performed for 596 (97.7%) and 79 miRNAs passed our training eligibility criteria (Table 1). The median estimated cis-h^2^ among this subset was 0.151 and interquartile range (IQR) was [0.096, 0.233]. Similarly, for the TCGA tumor miRNA expression data, 560/569 (98.4%) had cis-h^2^ testing results and 48 met GReX training criteria (overlap with normal: 22). The median estimated cis-h^2^ among this subset was 0.143 (IQR = [0.106, 0.267]). Supplementary Figure S3 compares the cis-h^2^ estimates and corresponding Wald 95% confidence intervals for all miRNAs expressed in both datasets and where GReX models were trained in at least one dataset. A total of 77/79 normal tissue GReX models and 47/48 tumor tissue GReX models corresponded to at least one non-zero SNP weight and were considered for further miTWAS analysis, resulting in successful GReX model training proportions of 12.6 and 8.3% for the normal and tumor tissue data, respectively.

**TABLE 1:** Summary of miRNA GReX model training for tumor and normal expression datasets.

	Number of mature miRNAs			GReX trained models		
MiRNA source	Expressed^a^	Failed	Eligible for GReX^b^	Trained^c^	cis-h^2^	# SNPs
			N (%)	N (%)	Mean (range)	Mean (range)
Mayo (Normal)	610	14	79 (13%)	77 (12.6%)	0.154 (0.032, 0793)	27 (1, 82)
TCGA (Tumor)	569	9	48 (8.4%)	47 (8.3%)	0.144 (0.061, 0.598)	30 (4, 94)

a Expressed mature miRNAs were identified as ≥ 5 reads in >25% of samples. Excludes miRNAs in the MHC region.

b MiRNAs with estimated cis-heritability > 0 and heritability test *p*-value < 0.01 were considered eligible for GReX training.

c MiRNAs with at least one non-zero SNP weight in the final GReX model.

To provide relative context on miRNA training, we compared these GReX training results to those for protein-coding genes in our comparably sized normal prostate tissue RNA-Seq expression dataset. Among 16,592 expressed protein-coding genes, GReX models were successfully trained for 7,088 genes (43%), nearly 3-4X that of the respective miRNA data. Among genes with trained models, the distribution of cis-h^2^ estimates was comparable (median = 0.171; IQR = [0.112, 0.288]).

### MiRNA TWAS Analyses

TWAS analyses were performed on all unique miRNAs with successfully trained GReX models (77 Mayo, 47 TCGA), of which 22 were shared across datasets. Initial review of these results revealed a number of instances of miRNAs from the same miRNA cluster and identical TWAS output (e.g., hsa-miR-3910 and hsa-miR-3910.1). To reduce redundancy, results were reviewed for members of the same family and, if identical (i.e., same GReX model characteristics, same TWAS statistics), a representative finding was carried forward to the final set of results. This yielded 71 and 39 miTWAS results in the normal and tumor tissue datasets, respectively, with 18 expressed miRNAs shared. Comparison of the TWAS Z-statistics among the 18 miRNAs analyzed revealed fairly consistent findings across datasets (Supplementary Figure S4).

**FIGURE 1:**
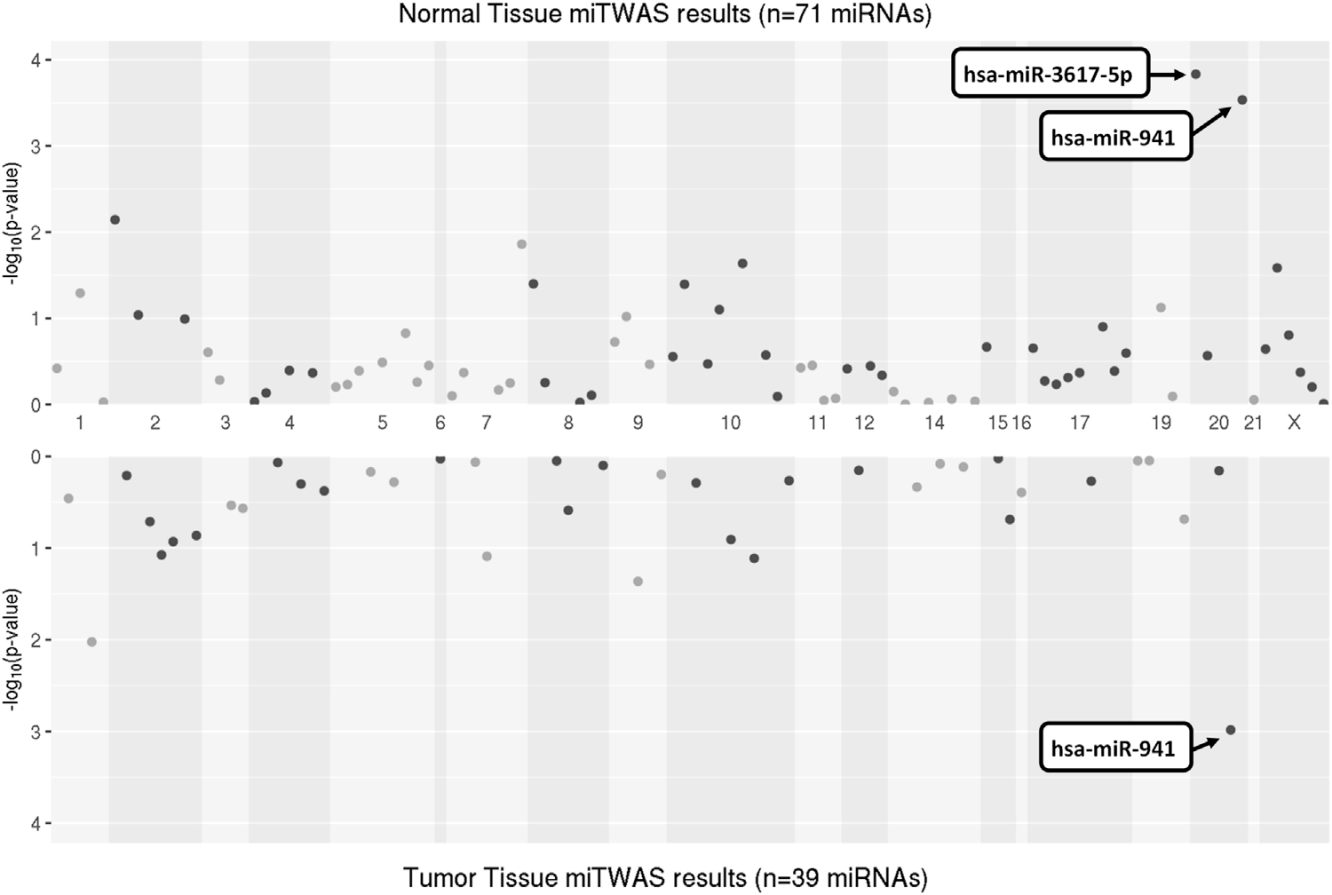
Hudson-style plot of miTWAS results, displaying−log10(P) for miRNA GReX models trained on eQTL datasets from Mayo normal prostate tissue (top) and TCGA PRAD tumor tissue (bottom). Significant results based on FDR<0.05 are labeled by miRNA.

Using the FDR< 0.05 significance criterion, we identified two significant miTWAS results on chr20 in at least one expression dataset (Table 2): hsa-miR-941 (P_normal_ = 2.9e-04 in normal; P_tumor_ = 1.0e-03) and hsa-miR-3617-5p (P_normal_ = 1.5e-04; not expressed in tumor). A Hudson plot of the complete miTWAS results across the Mayo normal and TCGA tumor prostate samples is presented in Figure 1, while complete TWAS results across both datasets are presented in Supplementary Table S1.

**TABLE 2:** MiTWAS results for miRNAs identified as significant (FDR<0.05) in at least one miRNA expression dataset.

	TWAS results								
Dataset	Mature miRNA	MiRNA Gene	Chr	Start (hg19)	cis-h2	Best GWAS SNP	Z	*p*-value	Q-value
Normal	hsa-miR-3617-5p	*MIR3617*	20	44333789	0.323	rs432448 chr20:44332298.G.T	−3.80	1.47E-04	0.0103
	hsa-miR-941	*MIR941*	20	62551155	0.793	rs1058319 chr20.62374389.C.T	3.62	2.92E-04	0.0103
Tumor	hsa-miR-941	*MIR941*	20	62551155	0.267	rs1058319 chr20.62374389.C.T	3.28	1.04E-03	0.0406

Top miTWAS result hsa-miR-3617-5p was evaluated solely in normal tissue (not considered expressed in tumor data), with the TWAS Z-score = −3.80 indicating reduced miR-3617-5p expression associated with increased PrCa risk. The best GWAS PrCa risk SNP result reported by FUSION was rs432448 (chr20:44332298.G.T), located approximately 1.5 kb from the *MIR3617* gene stem-loop sequence.

The miRNA hsa-miR-941 corresponded to a notably high expression level heritability in the Mayo normal tissue (cis-h^2^ = 0.79), which was similarly well-captured by the trained GReX model (cross-validated *R* ^2^ = 0.71). The top GWAS PrCa risk SNP was consistent across miRNA expression datasets (rs1058319; chr20.62374389.C.T). TWAS Z-scores were also consistent in direction across datasets (TWAS Z_normal_ = 3.62, TWAS Z_tumor_ = 3.28), indicating higher predicted miR-941 expression associated with increased PrCa risk. The mature miRNA originates from one of five tightly clustered stem-loop sequences within a ∼500 bp region, located at the 62.5 Mb region of chr20, approximately 177 kb from rs1053819.

### TWAS Finemapping Results

We first examined previously reported multi-tissue PrCa risk TWAS results by Mancuso et al. (Mancuso et al., 2018) for protein-coding mRNAs to determine if associations had been previously identified in proximity to (i.e., within 1 Mb) hsa-miR-941 and hsa-miR-3617-5p. For hsa-miR-941, multiple results were reported for a variety of genes and source tissues about the 62.3 Mb region of chr20, with the best GWAS SNP also identified for the majority of results to be rs1058319. The top overall result was *ARFRP1* in CMC. BRAIN.RNASEQ (TWAS *p* = 2.03E-20), while the top prostate-specific result was *ZGPAT* in the TCGA PRAD tumor dataset (TWAS *p* = 2.5E-11). No results were reported in proximity to miR-3617-5p.

Finemapping results from FOCUS for chr20 encompassed the two significant miRNA discoveries from our miTWAS analyses. The corresponding fine-mapping regions were chr20:42680754–44838826 (hsa-miR-3617-5p) and 20:62190180–62964897 (hsa-miR-941). The null model *p*-value for the hsa-miR-3617-5p region was the leading result, with a posterior inclusion probability (PIP) of 0.078. The PIP for hsa-miR-3617-5p was 0.0117, with no strong result for any given gene (leading gene result: *L3MBTL1* from brain in GTEx with PIP = 0.054). The 90% credible set was also relatively large (345 GReX models), which included hsa-miR-3617-5p. For the hsa-miR-941 region, a total of 10 genes comprised the 90% credible set, with the leading result corresponding to *DIDO1* from the brain_cortex in GTEx (PIP = 0.658). No prostate tissue GReX models were included in the credible set. Complete FOCUS output for the two relevant loci is presented in Supplementary Table S2.

## Discussion

In this study, we leveraged large prostate normal and tumor small RNA sequencing datasets to explore miRNA-based TWAS associations with genetic risk of PrCa and identified two significant associations: miR-941 and miR-3617-5p. Through the GReX training process, we observed a substantially lower overall proportion of miRNAs with successfully trained expression prediction models relative to protein-coding genes. This may be attributable to both biological and technical factors. First, the absolute expression levels were generally much lower in our normal tissue miRNA expression dataset than our much deeper sequenced long RNA-Seq data, which may reduce heritability testing power and predictive modeling performance. Moreover, there is evidence that the *cis*-regulatory contributions to overall heritability for miRNAs may be relatively modest compared to mRNAs. In the large multi-generational miRNA expression analysis of N = 5,239 Framingham Heart Study participants by Huan et al. (Huan et al., 2015), only 9/247 (3.6%) of expressed miRNAs corresponded to a narrow-sense h^2^ estimate >0.3, of which the mean single *cis*-eQTL contribution to explained variation was 0.08. As miRNAs are also regulatory non-coding RNAs, they are subject to sponging effects of target mRNAs and may be more susceptible to overall transcriptomic variability. Finally, as similarly speculated in Huan et al., it is likely that evolutionary pressures may constrain *cis*-regulatory effects on pri-miRNAs, given the roles of miRNAs on critical biological processes.

We also noted a sizable difference in number of trained models by dataset (77 for normal vs. 47 for tumor). We believe this may be again driven by both biological and technical differences between datasets. Firstly, the microRNA transcriptome can be heavily dysregulated in the tumor environment, which substantially increases the variance of expression and reduces the relative contribution of *cis*-acting genetic effects. Second, the relative density of the respective genotyping platforms could yield differential imputation efficiency for key eQTL SNPs, potentially yielding more accurate GReX models derived from the normal tissue samples. Despite these caveats, we did observe consistent results across the independent tumor and normal tissue miRNA expression datasets, both with respect to identified expressed miRNAs and miTWAS test statistics.

Our top miTWAS finding was miR-3617-5p, encoded by *MIR3617*, identified in the normal prostate tissue analysis. This miRNA was only identified as expressed in normal prostate tissue, with reduced levels corresponding to increased PrCa risk. Upon literature review, little is currently known regarding the biology of miR-3617-5p and its potential involvement to tumorigenic processes. A previous study of small-cell lung cancer identified downregulation of miR-3617-5p to be associated with chemoresitance (Kuang et al., 2020).

The miR-941 gene family consists of a cluster of five miRNA sequences spanning approximately 500 bp on chr20 (bp: 62550778–62551292), and hsa-miR-941 upregulation was identified to be associated with increased PrCa risk in both tumor and normal datasets. Hsa-miR-941 is a human-specific miRNA that is expressed in a wide variety of tissue types, and has demonstrated higher expression levels in cancer-derived cell lines (Hu et al., 2012). Hu et al. identified tumor suppressor lncRNA *TP73-AS1* as a sponge transcript for hsa-miR-941, showing that over-expression of TP73-AS1 attenuated cell migration and led to increased expression of hsa-miR-941 targets (Hu et al., 2018). Zhao et al. (2020) investigated serum exosomal miR-941 levels in laryngeal squamous cell carcinoma, identifying increased expression as a diagnostic biomarker. The authors further demonstrated miR-941 overexpression promoted cell proliferation and invasion via cell-line studies. These findings are commensurate with the observed positive association with PrCa risk in our study.

The relatively modest miTWAS associations along with our finemapping results raise questions regarding the potential causal relationship of PrCa genetic risk mediated by direct expression dysregulation of these two miRNAs and, to a larger extent, the prostate miRNA transcriptome in general. Similarly, while both identified miRNAs have previously reported associations with cancer in the literature, this is true of many miRNAs and thus such connections should be interpreted with caution. Consequently, follow-up functional studies that can study the direct effects of up/down-regulation of these miRNAs in prostate cell-lines are warranted.

There are limitations to our study that warrant mention. Firstly, no true replication dataset was available to verify discoveries, as the two datasets we analyzed were from normal, and tumor prostate tissue, respectively. Thus, further validation of these findings is warranted in independent and comparable datasets. Additionally, the high degree of sample multi-plexing in the normal tissue small RNA sequencing may have reduced sensitivity for association analysis with lower expressed mature miRNAs. However, we observed a high degree of overlap in identified miRNAs, and the individual miTWAS results across datasets were comparable. Our analyses were also restricted to subjects of European ancestry, which limits the generalizability of our results to other racial/ethnic groups. Finally, TWAS methods focus solely on *cis*-regulatory effects of trait-associated SNPs on expression. As miRNAs are themselves regulatory in nature, *trans*-effects of PrCa risk variants may be more relevant via competing endogenous RNA (ceRNA) networks.

In conclusion, our exploration of prostate miRNA expression and PrCa risk genetics revealed two moderate miTWAS associations, including an association in one region with no previously reported TWAS associations. Future efforts studying the role of miRNAs in the genetic risk of PrCa will focus on more sophisticated examination of ceRNA-based trans-effects and multi-tissue analyses.

## Data Availability

The data presented in the study are deposited in the dbGaP repository, accession number phs000985.v2.p1. Additional data from TCGA are publicly accessible and were downloaded from the GDC.
