# Supplementary material for: A microRNA Transcriptome-wide Association Study of Prostate Cancer Risk
Source: Front Genet. 2022 Mar 30;13:836841. doi: 10.3389/fgene.2022.836841 (PMC9006872; doi:10.3389/fgene.2022.836841)
Supplement: Supplementary file 3 [file DataSheet1.DOCX]

Supplementary Material

# Supplementary Figures and Tables

There are two (2) supplementary tables (separate files) and four (4) supplementary figures.

## Supplementary Tables

**Table S1 (TableS1.xlsx):** Complete miRNA TWAS results for the respective Mayo and TCGA datasets. Column headings are based on standard FUSION output column naming.

**Table S2 (TableS2.xlsx):** FOCUS TWAS finemapping results for two hit loci on chr20.

## Supplementary Figures


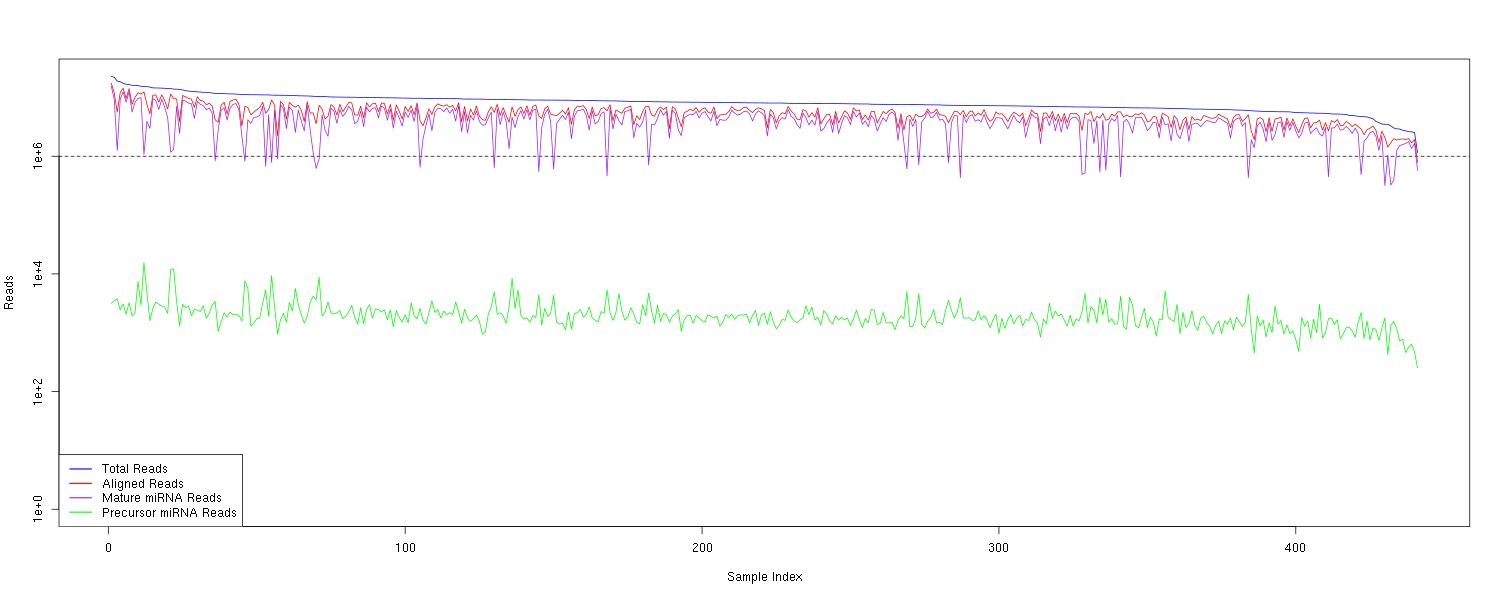


**Figure S1**: Small RNA sequencing output in terms of read counts (y-axis) summarized across all 441 QC-passed normal tissue prostate samples (x-axis). Separate lines indicate sequencing output separated by total reads (blue), aligned reads (red), reads aligned to mature miRNAs (purple), and precursor miRNA reads (green). Read count (y-axis) is presented under a log scale (horizontal dotted line indicates 1M reads for reference).

**
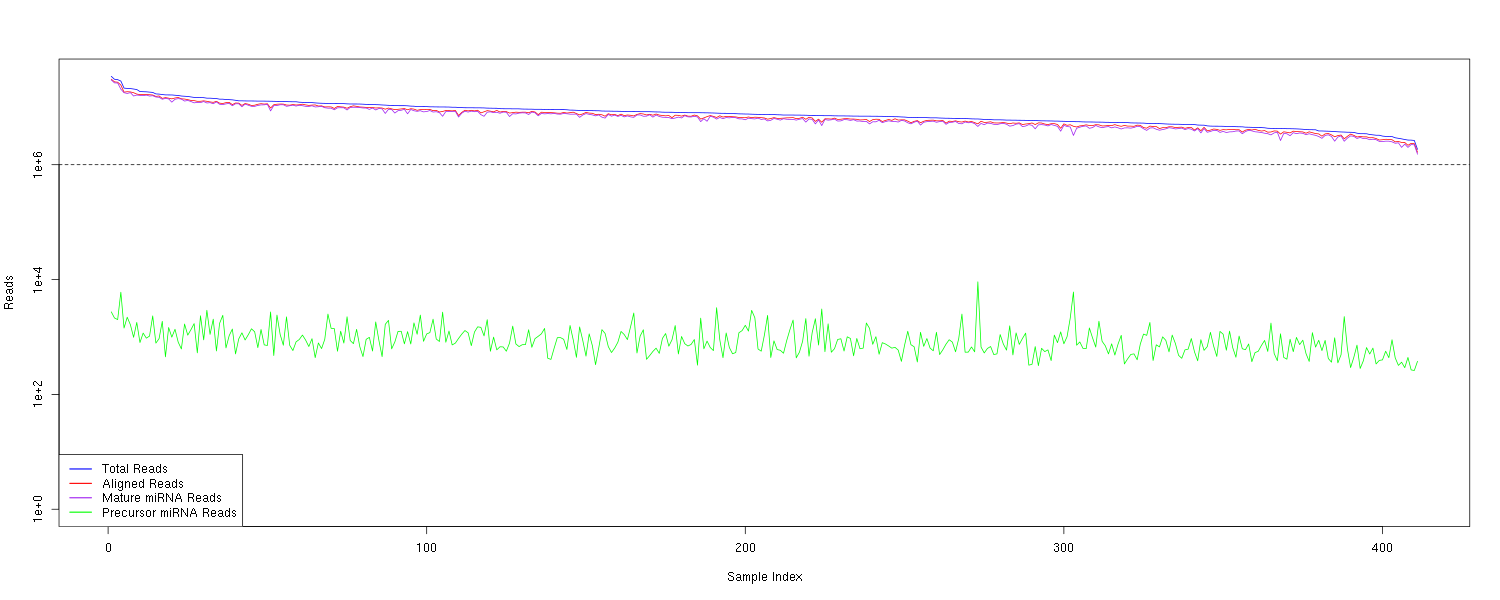
**

**Figure S2**: Small RNA sequencing output in terms of read counts (y-axis) summarized across all 411 TCGA tumor tissue prostate samples (x-axis). Separate lines indicate sequencing output separated by total reads (blue), aligned reads (red), reads aligned to mature miRNAs (purple), and precursor miRNA reads (green). Read count (y-axis) is presented under a log scale (horizontal dotted line indicates 1M reads for reference).


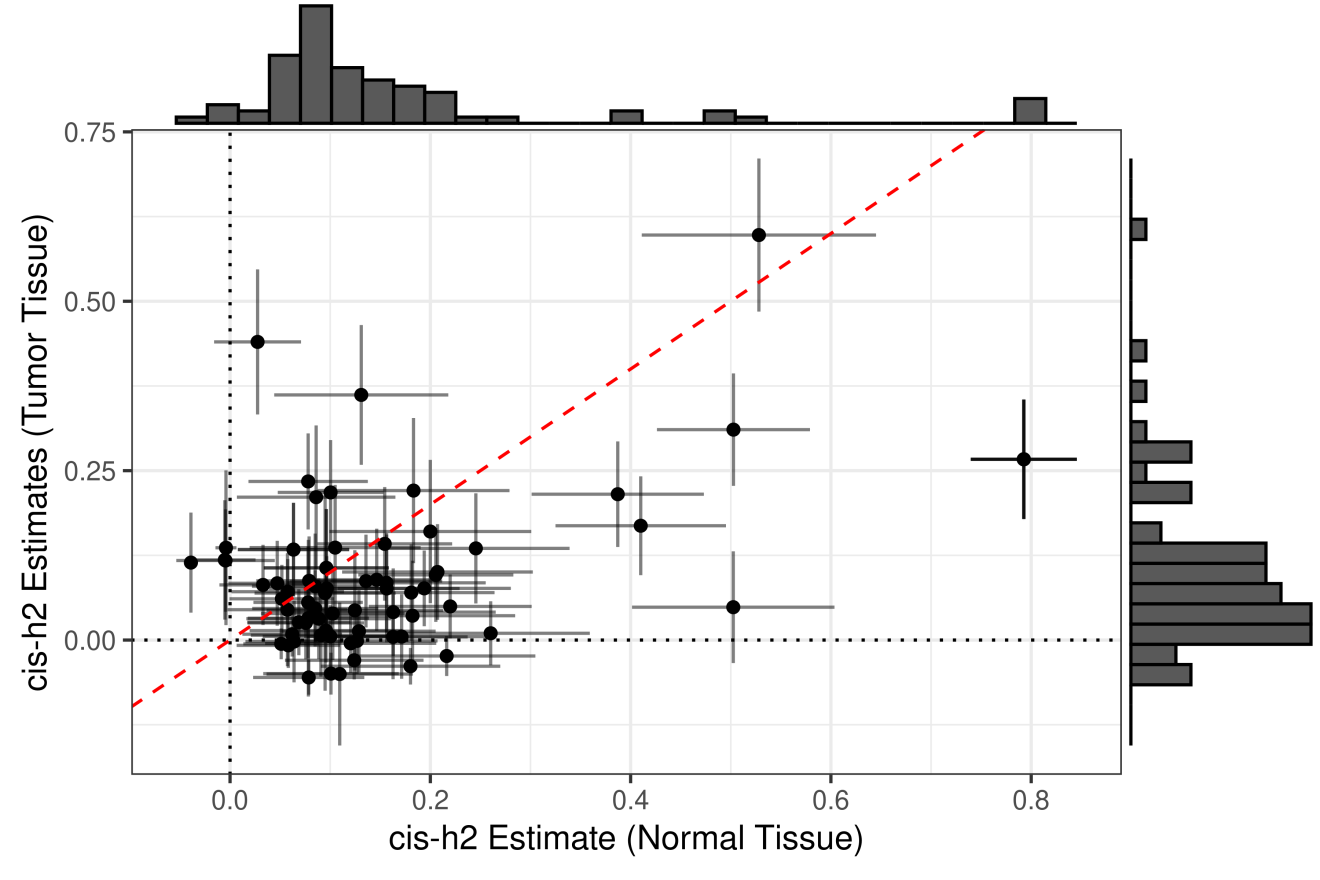


**Figure S3**: Scatterplot comparing miRNA cis-h^2^ estimates from normal prostate tissue (x-axis) and tumor (y-axis) among 74 overlapping miRNAs. Respective 95% confidence intervals are indicated by the overlapping crosses, and marginal distributions for each are depicted as histograms.


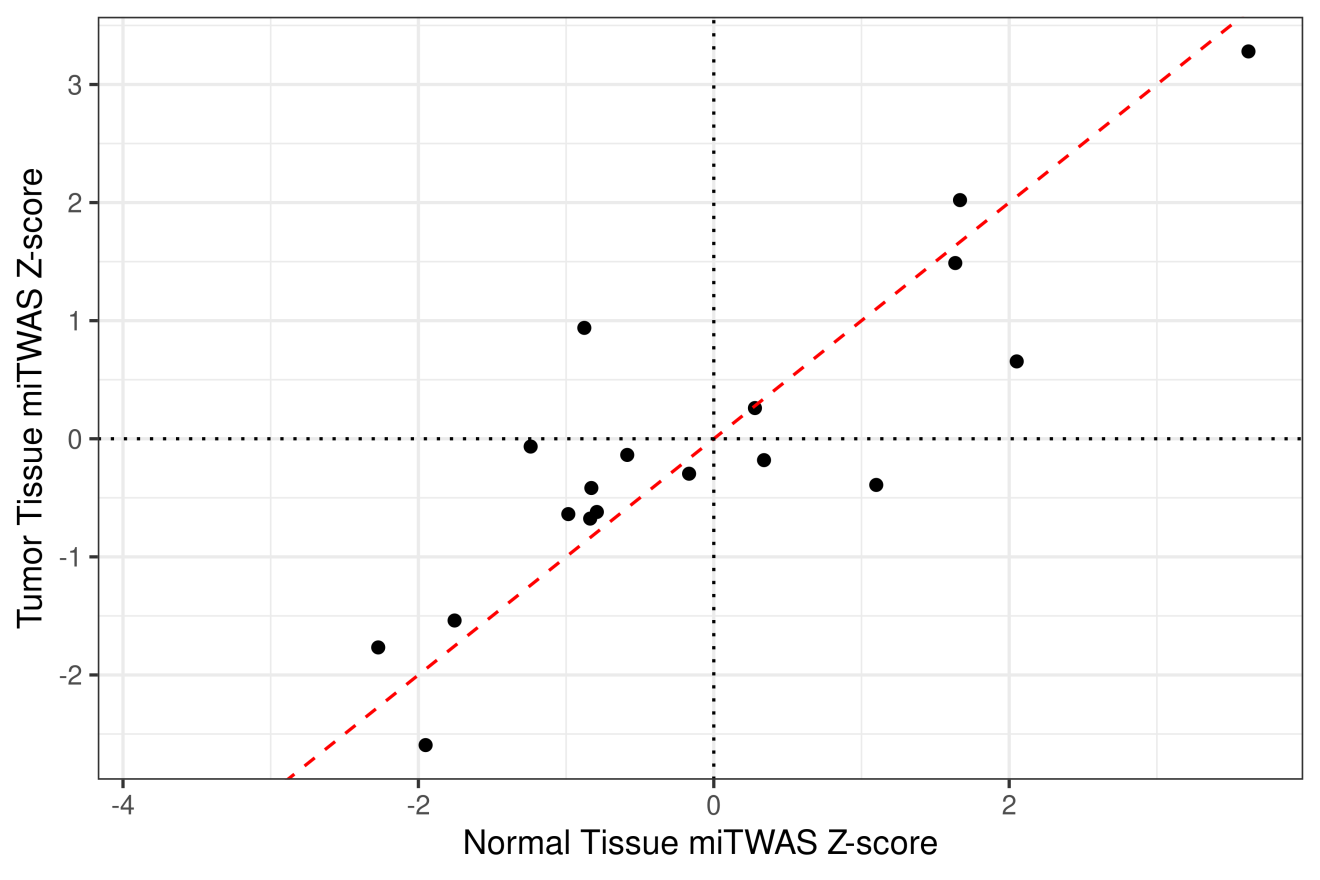


**Figure S4**: Scatterplot of TWAS Z-statistics for 18 overlapping miRNAs in the normal and tumor prostate tissue datasets eligible for analysis.
